# Identifying policy-relevant traffic crash risk factors in Cheongju, South Korea using logistic regression and explainable machine learning

**DOI:** 10.1371/journal.pone.0350616

**Published:** 2026-06-22

**Authors:** Eun-Ji Lee, Sua Kim, Hyun-Ji Lee, Jae-Hwan Jhong

**Affiliations:** 1 Department of Statistics, Chung Buk National University, Cheongju, Chungbuk, Republic of Korea; 2 The Korea Transport Institute, Sejong-si, Sejong, Republic of Korea; Tongji University, CHINA

## Abstract

Rapid urbanization and increasing traffic volumes have made the occurrence of traffic crashes and the resulting harm a major public safety concern. This study analyzes traffic crash data from Cheongju, a mid-sized city in Chungcheongbuk-do, to identify key determinants of crash severity and provide evidence-based policy recommendations. Our approach is novel in that it integrates statistical modeling and machine learning methodologies; this dual approach not only overcomes the limitations inherent in using either technique alone but also allows for the identification of consistent risk factors influencing traffic crash severity that may have gone unrecognized otherwise. Marginal effects of explanatory variables were interpreted using ordinal logistic regression, while feature importance in machine learning models—including Support Vector Machine, Random Forest, XGBoost, and LightGBM—was evaluated using SHAP (SHapley Additive exPlanations) values. Both analytical approaches consistently identified traffic signal violations, failure to comply with safe driving obligations, and the absence of a median barrier on undivided roads as significant predictors of crash severity. By leveraging empirical data specific to Cheongju, our research provides regionally tailored insights that distinguish our work from prior studies with broader or less localized focus. These findings highlight the need for stricter enforcement of traffic regulations and structural improvements in roadway infrastructure and can inform policymakers in formulating effective, context-specific measures to enhance road safety.

## Introduction

Developing effective traffic safety policies requires a solid scientific understanding of traffic crash severity and its associated risk factors, particularly in complex and heterogeneous traffic environments. The use of data analytics is essential in this context, as systematic analyses of crash severity play a critical role in informing evidence-based safety strategies. However, in many small to mid-sized cities in South Korea, data-driven approaches to traffic safety policy remain underdeveloped, limiting the ability to design practical and context-sensitive safety interventions.

In particular, comprehensive traffic crash analyses that jointly consider local characteristics—such as road conditions, traffic flow, and population demographics—are still insufficient. This lack of detailed and systematic evidence constrains the understanding of crash severity and its key risk factors, thereby hindering the formulation of effective, localized traffic safety measures and the weakening of the scientific basis for policymaking.

These challenges are especially pronounced in mid-sized cities with mixed urban–rural structures. In such settings, urban and rural zones coexist, giving rise to heterogeneous road infrastructures, population structures, and traffic patterns, which in turn produce diverse crash scenarios. Addressing these complexities requires customized analytical approaches grounded in local empirical data rather than uniform policy frameworks.

Cheongju, a mid-sized urban–rural city located in the central region of South Korea, exemplifies these conditions. It is one of the largest cities in the country outside of the designated metropolitan areas and is notable for experiencing a steady year-on-year population increase, in contrast to the broader national trend of population decline. The city encompasses a wide range of traffic environments, including single roads, intersections, and mixed-use streets, and has experienced a continuous rise in traffic volume driven by ongoing urbanization and population growth. Despite these trends, the expansion of transportation infrastructure and the development of traffic safety policies have not kept pace, and detailed, systematic analyses of traffic crash severity that reflect local characteristics remain limited. In this context, Cheongju provides an appropriate case study for examining diverse traffic crash scenarios and for generating empirically grounded insights that can support the development of targeted and actionable traffic safety policies applicable to other mid-sized cities with similar characteristics.

To situate this study within the broader literature, we organize existing research into three lines according to their primary methodological orientation and analytical focus.

The first line comprises studies that apply statistical models to identify risk factors associated with crash severity. [1,2] explored methods to quantify traffic risks and externalities using sophisticated models and optimization approaches. [3] employed a fixed-effects panel model to examine the effects of climate change, socioeconomic factors, and regulations on fatal traffic crashes. Several studies have employed ordinal logistic regression to analyze determinants of crash severity ([4–9]). Notably, [7] developed a spatio-temporal logistic regression model that accounts for spatial and temporal correlations to identify significant predictors of pedestrian injury severity, and [10] conducted in-depth analyses using statistical models to examine the relationship between injury severity, roadway characteristics, and driver attributes. While these statistical approaches offer clear interpretability through coefficients and marginal effects, they are inherently constrained by linearity assumptions and limited capacity to capture complex interactions among variables.

The second line encompasses studies that apply machine learning algorithms to predict crash severity. [11] applied XGBoost to investigate accidents involving vulnerable road users in China, while [12–18] applied various machine learning techniques to predict injury severity in traffic crashes. [19] compared sampling methods to address class imbalance in crash severity prediction, and [20] investigated behavioral pathways affecting police officer injury severity using collision data. These machine learning approaches demonstrate strong predictive performance by flexibly capturing nonlinear relationships and high-order interactions. However, their application has been predominantly oriented toward maximizing predictive accuracy, with relatively limited attention paid to interpreting the direction, magnitude, and policy relevance of individual risk factors.

The third line consists of studies that integrate both statistical and machine learning approaches. [21,22] employed both logistic regression and machine learning techniques, comparing their predictive performance and explanatory capabilities to identify critical factors influencing crash severity. While this combined use of methods represents a step forward, these studies remain primarily focused on comparing predictive accuracy across models. The interpretation of results tends to be conducted separately within each methodological framework, and a systematic, complementary integration of the inferential strengths of statistical models with the flexibility of machine learning—specifically for the purpose of policy-relevant factor identification—has received insufficient attention in the literature.

This gap motivates the present study. Unlike studies that frame crash severity analysis as a prediction problem, this study is fundamentally oriented toward explanation and interpretation: identifying key risk factors influencing traffic crash severity and interpreting both the direction and magnitude of their effects in a policy-relevant manner. To this end, we apply ordinal logistic regression and four tree-based machine learning algorithms, and interpret their outputs through complementary analytical lenses: marginal effects for the statistical model and SHAP (SHapley Additive exPlanations) values for the machine learning models. This dual interpretive framework offers distinct but mutually reinforcing perspectives—marginal effects capture average directional impacts across the sample, while SHAP values reveal relative feature importance and effect heterogeneity across crash severity levels. By cross-validating findings across these two frameworks, the study is able to identify robust and consistent risk factors that might otherwise remain undetected when relying on a single modeling approach.

Furthermore, the study grounds its analysis in local empirical data from Cheongju, enabling the generation of context-specific evidence that reflects the city’s unique urban–rural complexity. This localized focus not only enhances the practical relevance of the findings but also contributes to filling an important gap in the traffic safety literature on mid-sized Korean cities, where systematic, data-driven analyses remain scarce.

The remainder of this paper is organized as follows. The Data section outlines the data collection, preprocessing procedures, and exploratory data analysis. The Methods section introduces the methodological framework, including the ordinal logistic regression model and four machine learning algorithms. The Results section presents the empirical findings based on traffic crash data from Cheongju. Finally, the Discussion and Conclusion sections address the main conclusions, limitations, and implications for future research and policy.

## Data

This section describes the data collection and preprocessing procedures. Using the statistical software package R, it presents the results of descriptive statistical analysis, multicollinearity diagnostics, and correlation analysis among the explanatory variables.

### Data collection

This study utilized traffic crash data from Cheongju, Chungcheongbuk-do, to analyze factors affecting crash severity, covering the period from 2019 to 2023 based on police-reported traffic crash. The data were sourced from the Traffic Accident Analysis System (TAAS, https://www.koroad.or.kr/) operated by the Korea Road Traffic Authority. The dataset includes five years of traffic crash records in Cheongju, encompassing fatal crash, serious injuries, minor injuries, and reported injury crash. TAAS provides comprehensive traffic accident-related information such as the date and details of the crash, traffic violations, road surface and weather conditions, road types, as well as information on both the at-fault and victim drivers.

### Data pre-processing

The response variable used to identify risk factors affecting traffic accident severity is the *severity*. In TAAS dataset, human casualties resulting from traffic crash are classified into four severity levels: Injury, Minor, Serious, and Death, which are defined in Table 1. Note that the response variable for severity was defined based on the most severe injury among the involved individuals. These classifications are based on the duration of medical treatment required and the outcome of the crash. The system utilizes these categories to analyze crash data, assess road safety, and formulate policies aimed at reducing traffic-related injuries and fatalities.

**Table 1:** The description of *severity* levels.

Level	Description
Injury	Injuries requiring medical treatment for less than 5 days due to a traffic crash
Minor	Injuries requiring medical treatment
	for 5 days or more but less than 3 weeks due to a traffic crash
Serious	Injuries requiring medical treatment for 3 weeks or longer due to a traffic crash
Death	Death occurring within 30 days from the date of the traffic crash

To analyze the factors influencing traffic accident severity, [14] considered 12 variables including *violation* and *season*, which were categorized into crash factors, environmental factors, road factors, vehicle factors, and human factors. In this study, crash factors include the number of crash (*count*) and types of traffic violations (*violation*). Environmental factors comprise season (*season*), day of the week (*weekday*), and weather conditions (*weather_condition*).

Road factors consist of road type (*road_type*), vehicle factors include the type of at-fault vehicle (*perpetrator_car*), and human factors consider the at-fault driver’s gender (*perpetrator_gender*) and age group (*perpetrator_age*), resulting in a total of nine explanatory variables. Among the crash factors, the *count* variable is a derived metric representing the total number of casualties by summing fatalities, serious injuries, minor injuries, and reported injuries.

For environmental factors, the *season* variable was created by extracting the month from the crash date and classifying months 3–5 as spring, 6–8 as summer, 9–11 as autumn, and 12–2 as winter. The *weekday* variable was binarized based on the day of the week, coding Monday through Friday as weekdays (1) and Saturday and Sunday as weekends (0). Regarding human factors, the *perpetrator_age* variable was recoded as a categorical variable grouping ages under 20 by combining those 12 years and younger with those aged 13–20, coded as 20. Subsequent age groups were coded using representative numbers, such as 21 for drivers in their 20s, 31 for those in their 30s, and so forth. Note that although numeric labels were used to denote age-group categories for illustrative purposes, perpetrator_age was ultimately treated as a categorical variable and encoded using one-hot encoding, ensuring that no ordinal or numerical structure was assumed.

Since speeding-related crash began to be separately recorded only from 2021, any speeding violations in the 2019 and 2020 data were reclassified as “Other” within the violation category. Records with missing or “Other” entries in the variables *perpetrator_gender*, *perpetrator_age*, *violation*, and *weather_condition* were excluded from the analysis. After this preprocessing, a total of 19,071 crash cases were included in the final analysis. S1 Table summarizes the composition and descriptions of the variables used in the final analysis.

### Exploratory data analysis

To understand the overall distribution of the variables, descriptive statistics were reviewed. S1 Table presents descriptive information for each variable. Regarding the response variable *severity*, Minor accounted for the largest proportion (70.46%), followed by Serious (25.82%), Injury (2.64%), and Death (1.08%). Only 3.72% of all cases fell into the Injury and Death categories, indicating a substantial class imbalance in traffic accident severity.

Among traffic accident-related factors, the variable *count* showed an average of approximately 1.5 casualties per crash, with *violation* most frequently classified as failure to drive safely. Environmental factors such as *season* were evenly distributed, while the majority of crash occurred on weekdays and under clear weather conditions. Regarding road and vehicle characteristics, most crash occurred at crossroads and involved passenger cars. In terms of human factors, male drivers constituted the majority of at-fault individuals, with a substantial proportion being middle-aged or older.

S2 Table presents the generalized variance inflation factor (GVIF) values for all explanatory variables. GVIF is used to detect multicollinearity, particularly when models include categorical variables with multiple levels. All GVIF values were below the commonly accepted threshold of 5, indicating no serious multicollinearity among the variables.

In addition, an association analysis was conducted to examine whether each explanatory variable had a statistically significant effect on the response variable. For the continuous variable *count*, a one-way analysis of variance (ANOVA) was applied, while chi-square tests were performed for the categorical explanatory variables.

S3 Table summarizes the results of the association analysis between each explanatory variable and the response variable. The results showed that all explanatory variables except for *weekday* exhibited statistically significant associations with the response variable at the 5% significance level. Although *weekday* was not statistically significant, it was retained in the analysis in consideration of the study’s primary objective—identifying factors influencing the severity of traffic crash—rather than merely predicting severity. The variable was included to allow for meaningful social interpretation and potential policy implications.

## Methods

### Ordinal logistic model

The ordinal logistic regression model is a commonly used statistical method for analyzing ordinal categorical response variables. It is particularly well-suited for modeling traffic crash severity, which is inherently ordered—ranging from minor injury to death—as it explicitly accounts for the ordinal structure of the response rather than treating categories as nominal or continuous.

A widely adopted specification is the proportional odds model, defined as:

$$\begin{array}{c} \log\left(\frac{P(Y\leq k)}{P(Y>k)}\right)=\alpha_{k}-X^{⊤}\beta ,\;\text{for }k=1,\ldots ,K-1, \end{array}$$ (1)

where *Y* is the ordinal response variable with *K* ordered categories, $\alpha_{k}$ is a threshold (cut-point) parameter for category *k*, $X=(X_{1},\ldots ,X_{J}{)}^{⊤}$ is the vector of explanatory variables with *J* denoting the number of explanatory variables, and $\beta =(\beta_{1},\ldots ,\beta_{J}{)}^{⊤}$ is the coefficient vector.

This model assumes the proportional odds (parallel slopes) assumption [23], meaning the effect of X is consistent across cumulative logits. While coefficients β are often interpreted via odds ratios, they represent multiplicative changes in odds and do not directly convey how the predicted probability of each severity category changes with a given covariate. This limits their interpretability in policy contexts, where understanding the magnitude of probability changes is more actionable than relative odds. While most existing studies employing logistic regression have relied on odds ratios for interpretation, this study adopts average marginal effects (AME) to provide a more direct and policy-relevant measure of each risk factor’s influence on crash severity outcomes.

To address this, we use average marginal effects (AME), which represent the average change in the predicted probability of a specific response category *k* when an explanatory variable changes, holding all others constant.

For continuous variables $X_{j}$, where j=1,…,J indexes explanatory variables and *N* is the total number of observations, the marginal effect for observation *i* is:


$$\begin{array}{c} {\text{ME}}_{ij}^{\left(k\right)}=\frac{\partial P(Y_{i}=k|X_{i})}{\partial X_{ij}},\;{\text{AME}}_{j}^{\left(k\right)}=\frac{1}{N}\sum_{i=1}^{N}{\text{ME}}_{ij}^{\left(k\right)}. \end{array}$$

For categorical variables $C_{j}$ with baseline *c*_1_ and comparison $c_{m}$, where $X_{i,-j}$ denotes all covariates except $C_{j}$ for observation *i*:


$$\begin{array}{c} {\text{ME}}_{ij,m}^{\left(k\right)}=P(Y_{i}=k|C_{ij}=c_{m},X_{i,-j})-P(Y_{i}=k|C_{ij}=c_{1},X_{i,-j}), \end{array}$$

$$\begin{array}{c} {\text{AME}}_{j,m}^{\left(k\right)}=\frac{1}{N}\sum_{i=1}^{N}{\text{ME}}_{ij,m}^{\left(k\right)}. \end{array}$$

Unlike odds ratios, AMEs are expressed in probability units, making them directly interpretable: a positive AME for a given variable at a given severity level indicates that the variable increases the predicted probability of that outcome, with the magnitude reflecting the size of the effect. Furthermore, by construction, the sum of AMEs across all response categories equals zero, ensuring internal consistency, and larger absolute values imply greater influence on the response. This probability-based interpretation facilitates meaningful comparisons across severity levels and variables, and provides a more intuitive basis for policy recommendations.

### Machine learning algorithms

#### Algorithmic approach.

This study applies four representative machine learning algorithms— Support Vector Machine (SVM), Random Forest (RF), Extreme Gradient Boosting (XGBoost), and Light Gradient Boosting Machine (LightGBM)— to complement the ordinal logistic regression model. While the logistic regression model provides interpretable marginal effects under linearity assumptions, these machine learning algorithms are employed to capture nonlinear relationships and complex interactions that may not be fully represented in the statistical model, thereby addressing the multi-class classification problem of traffic crash outcomes and identifying key factors influencing traffic accident severity through variable importance analysis.

Unlike ordinal logistic regression, these algorithms do not impose linearity assumptions or require a predefined functional form, allowing them to flexibly capture nonlinear relationships and complex interaction effects among variables. This flexibility makes them particularly appropriate for modeling traffic crash severity, where the relationships between risk factors and outcomes are unlikely to be purely linear or additive. However, this flexibility comes at the cost of reduced direct interpretability, which is addressed through an explainability method described in the Variable Importance section.

SVM ([24]) is a supervised learning-based classification method that finds a hyperplane that maximizes the margin between classes in a high-dimensional space. By utilizing kernel functions, it can effectively handle nonlinear classification problems. For multi-class classification, the One-vs-Rest strategy is commonly employed. SVM is particularly useful when the number of features is large relative to the number of observations, and its kernel-based approach makes it well-suited for capturing complex decision boundaries in heterogeneous traffic environments.

RF ([25]) is an ensemble learning technique based on bagging that constructs multiple decision trees and aggregates their predictions. Each tree is trained using a randomly selected subset of samples and variables, which helps prevent overfitting, reduce prediction variance, and improve overall predictive performance. Its ensemble nature also provides inherent robustness to noise and outliers in the data, making it appropriate for real-world traffic crash datasets that often contain irregular or imbalanced observations.

XGBoost ([26]) is an enhanced gradient boosting algorithm that builds a strong predictive model by sequentially combining weak learners. It incorporates a regularized objective function, a highly efficient tree structure, and various optimization techniques to achieve both high prediction accuracy and computational efficiency. The built-in regularization mechanism helps prevent overfitting, which is particularly important given the class imbalance present in the traffic crash severity data used in this study.

LightGBM ([27]) is a gradient boosting framework designed for large-scale data processing. It improves training speed and memory efficiency by adopting histogram-based binning and a leaf-wise tree growth strategy. In addition, it supports automatic handling of categorical variables and various regularization techniques, making it highly applicable to real-world scenarios. This automatic handling of categorical variables is particularly advantageous in this study, where many explanatory variables—such as road type, weather condition, and violation type—are categorical in nature.

Model implementation and training were conducted in Python 3.11.11 environment, with major libraries including scikit-learn (version 1.5.1), XGBoost (version 3.0.0), and LightGBM (version 4.6.0). To prevent overfitting and underfitting, 5-fold cross-validation was applied.

#### Data splitting and sampling strategy.

Traffic accident severity is not the target of prediction in this study, but rather the focus is on analyzing factors influencing severity. Accordingly, the data is split into a training set (80%, 15,256 samples) and a test set (20%, 3,815 samples) without a separate validation set. To address the severe class imbalance in the response variable (Injury: 402, Minor: 10,750, Serious: 3,939, Death: 165), we explored two training strategies: (1) imbalanced training, which preserves the original distribution and naturally reflects real-world occurrences but may bias the model toward majority classes, and (2) balanced sampling, which combines oversampling of minority classes (using SMOTE) and undersampling of majority classes to achieve a more even distribution, potentially improving minority class performance but risking overfitting, information loss, and distorted probability interpretation. In our experiments, oversampling increased “Injury” and “Death” classes to 2,000 samples each, while the “Minor” class was undersampled to 4,000 samples, resulting in a training set of 13,939 samples.

The results indicate that applying sampling led to modest changes in performance, but did not yield consistent or substantial improvements across models. This outcome may reflect the extreme imbalance in the response variable and potential distortions of the original data distribution introduced by resampling. Given that the primary objective of this study is not to maximize predictive accuracy but to interpret the relative importance and direction of contribution of influencing factors, we prioritized model interpretability and the preservation of the original data structure. Accordingly, the main text focuses on results obtained from the original data, while the results from the sampling-based analyses are provided in the Supplementary Material (S6–S7 Tables).

#### Hyperparameter tuning.

To optimize model performance, key hyperparameters were tuned for each algorithm. Table 2 presents the key hyperparameters and their tuning ranges for four machine learning algorithms: SVM, Random Forest, XGBoost, and LightGBM. For SVM, parameters such as the regularization strength, kernel type, and gamma are adjusted to control the flexibility of the decision boundary and generalization performance. In Random Forest, the number of trees, maximum depth, and criteria for node splitting and leaf size are tuned to balance model complexity and prevent overfitting. XGBoost and LightGBM, both tree-based boosting models, optimize predictive accuracy and training efficiency by tuning parameters such as the number of trees, tree depth, learning rate, and sampling ratios. These tuning processes are typically conducted through grid search and cross-validation, serving as essential steps to enhance model performance.

**Table 2:** Tuning parameter performance optimization range.

Algorithm	Parameter	Value
SVM	C	0.1, 1, **10**
	kernel	linear, **rbf**
	gamma	**scale**, auto
RF	n_estimators	50, **100**, 200, 500
	max_depth	3, 5, 10, **20**
	min_samples_split	2, **5**, 10
	min_sample_leaf	**1**, 2, 4
XGBoost	n_estimators	50, 100, 200, **500**
	max_depth	3, **5**, 10, 20
	learning_rate	0.01, 0.05, 0.1, **0.2**
	subsample	0.4, 0.6, 0.8, **1.0**
LightGBM	n_estimators	50, 100, 200, **500**
	max_depth	3, **5**, 10, 20
	learning_rate	0.01, 0.05, **0.1**
	num_leaves	20, 30, **40**
	subsample	**0.6**, 0.8, 1.0
	colsample_bytree	0.6, 0.8, **1.0**

#### Performance evaluation.

To evaluate model performance, five metrics were used based on the test set: accuracy, precision, recall, macro F1-score, and weighted F1-score.

Accuracy indicates the proportion of correctly classified samples. Precision and recall respectively measure the correctness of positive predictions and the ability to detect true positives. The F1-score, the harmonic mean of precision and recall, is especially useful in imbalanced data settings. The macro F1-score averages F1-scores across all classes equally, while the weighted F1-score accounts for class imbalance by weighting each class by its sample size.

Let $TP_{k}$, $FP_{k}$, and $FN_{k}$ denote the true positives, false positives, and false negatives for class *k*, and $n_{k}$ the number of samples in class *k*. The metrics are computed as follows:


$$\begin{array}{c} \text{Accuracy}=\frac{\sum_{k=1}^{K}TP_{k}}{N},\;{\text{Precision}}_{k}=\frac{TP_{k}}{TP_{k}+FP_{k}}, \end{array}$$

$$\begin{array}{c} {\text{Recall}}_{k}=\frac{TP_{k}}{TP_{k}+FN_{k}},\;{\text{F1}}_{k}=\frac{2\cdot {\text{Precision}}_{k}\cdot {\text{Recall}}_{k}}{{\text{Precision}}_{k}+{\text{Recall}}_{k}}, \end{array}$$

$$\begin{array}{c} \text{Macro F1}=\frac{1}{K}\sum_{k=1}^{K}{\text{F1}}_{k},\;\text{Weighted F1}=\sum_{k=1}^{K}\frac{n_{k}}{N}\cdot {\text{F1}}_{k} \end{array}$$

#### Variable importance.

This study focuses on understanding the relative influence of explanatory variables rather than improving prediction accuracy, using SHapley Additive exPlanations (SHAP; [28]) values to identify variable importance. SHAP is a method based on cooperative game theory that intuitively and consistently quantifies each explanatory variable’s contribution to the model prediction.

SHAP values decompose the model prediction into a baseline value and the individual contributions of each variable. By averaging the contributions across all possible variable combinations, SHAP provides a fair and consistent assessment of variable importance. A key advantage of SHAP over traditional variable importance measures such as mean decrease in impurity and permutation-based importance, is that it provides both the direction and magnitude of each variable’s contribution to a specific predicted outcome, rather than merely ranking variables by aggregate importance. This allows for a nuanced interpretation of how each risk factor influences crash severity at each outcome level, which is particularly valuable in a policy context where understanding the nature of the effect—not just its existence—is critical.

Moreover, SHAP values naturally accommodate nonlinear relationships and interaction effects among variables, reflecting the complex structure captured by tree-based models. This makes SHAP a particularly appropriate tool for interpreting machine learning models in traffic safety research, where the relationships between risk factors and crash outcomes are unlikely to be purely linear or additive.

However, it should be noted that SHAP values represent model-based contributions to predicted outcomes and do not imply causal relationships. Interpretation should therefore be understood within the scope of the fitted model rather than as evidence of direct causal effects.

In this study, SHAP values were computed for the best-performing model selected through the evaluation procedure described above, using the TreeExplainer algorithm optimized for tree-based ensemble models. SHAP values were analyzed separately for each severity level (Injury, Minor, Serious, and Death), focusing on positive contributions to identify variables that increase the predicted probability of each severity outcome.

## Results

### Ordinal logistic regression model

An ordinal logistic regression model was employed to construct a model of traffic accident severity, and the marginal effects were derived accordingly. Marginal effects transform coefficients from a logistic regression model into changes in predicted probabilities, thereby providing clearer and more intuitive insights into how each explanatory variable influences each severity level of the response variable (*severity*)—namely, Injury, Minor, Serious, and Death.

**Fig 1:**
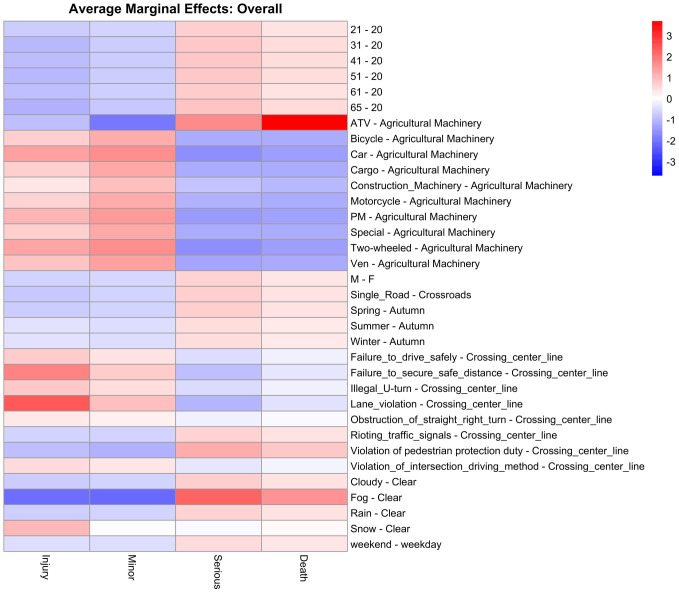
Heatmap of average marginal effects of all explanatory variables across traffic accident severity levels. Positive AMEs are shown in red and negative AMEs in blue; color intensity reflects the magnitude of the effect.

**Fig 2:**
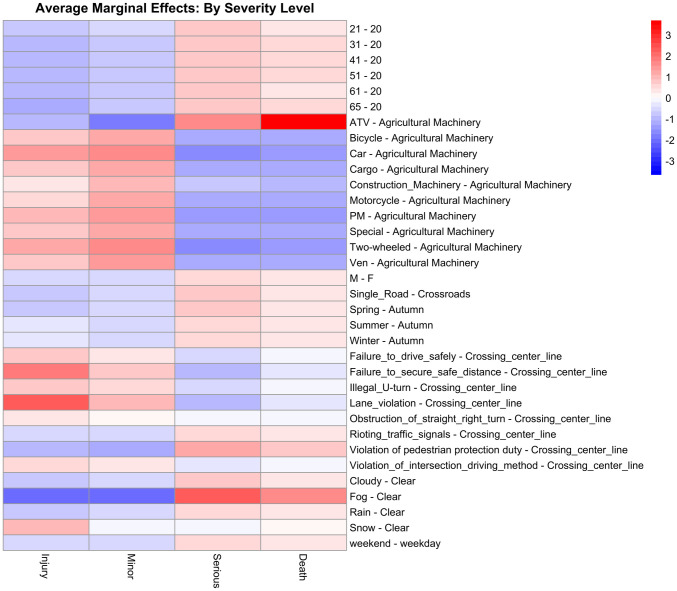
Heatmap of average marginal effects highlighting the most influential explanatory variables for each severity level. Positive AMEs are shown in red and negative AMEs in blue; color intensity reflects the magnitude of the effect.

**Fig 3:**
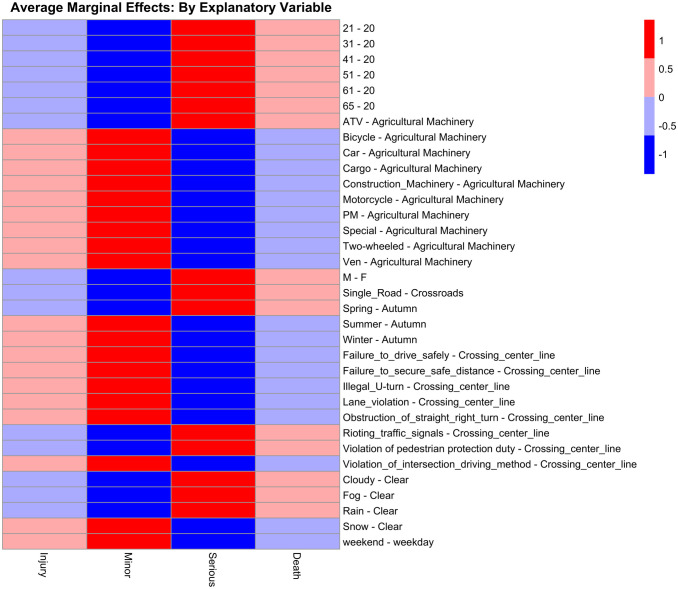
Heatmap of average marginal effects highlighting the most influenced severity level for each explanatory variable. Each color represents a distinct severity level (Injury, Minor, Serious, Death), highlighting which severity level is most strongly associated with each explanatory variable.

The average marginal effect values for each explanatory variable across the severity levels are summarized in S4 Table, and Figs 1–3 present a corresponding heatmap visualization. In the heatmap, positive estimates are shown in red and negative estimates in blue, allowing a clear visual comparison of the impact of each variable on different severity outcomes. In addition, the horizontal axis represents the level of the response variable, and the vertical axis represents the comparison of levels of the explanatory variables. On the other hand, while the main text focuses on marginal effects for interpretability, the full ordinal logistic regression results are reported in the Supplementary Material (S5 Table).

Fig 1 shows a heatmap of all 140 marginal effect values, corresponding to the 4 response variable levels multiplied by the 35 explanatory variables (or their levels), allowing a comprehensive comparison between response levels and explanatory variables at a glance. Fig 2 presents a heatmap using the same 35-color palette to clearly distinguish which explanatory variables (or their levels) affect each level of the response variable severity. Lastly, Fig 3 uses a 4-color palette to highlight which severity level is most influenced by each specific explanatory variable (or its level).

When comparing within each severity level, excluding Serious, the Injury, Minor, and Death categories appear to be strongly influenced by violations such as ‘Failure_to_drive_safely’, ‘Failure_secure_safe_distance’, ‘Illegal_U-turn’, Lane_violation’, ‘Violation_of_traffic_signals’, and ‘Violation_of_intersection_driving_method’, relative to ‘Crossing_center_line’. Additionally, for these severity levels, the probability of an crash was relatively higher under ‘Snow’ conditions compared to ‘Clear’ weather.

In contrast, the Serious category exhibits different patterns, being more influenced by the vehicle type compared to Agricultural ‘Machinery’, and particularly by adverse weather conditions such as ‘Fog’ rather than ‘Clear’.

When comparing AME values across severity levels, the results are generally consistent with those observed within levels. Legal violations tend to show large positive effects (displayed in red) for Injury, Minor, and Death levels, especially under snowy conditions, while their impact on Serious is relatively limited.

For Serious, the impact on traffic accident severity appears to vary more with vehicle type, and higher severity is also observed under weather conditions such as ‘Clear’, ‘Fog’, ‘Cloudy’, and ‘Rain’.

### Machine learning algorithm

Table 3 shows the performance evaluation for four machine learning algorithms. As a result of the final performance evaluation, the Random Forest model demonstrated the best classification performance. It achieved an Accuracy of 0.6975, Precision of 0.6220, Recall of 0.6975, a Macro-F1 Score of 0.2622, and a Weighted-F1 Score of 0.6237. In particular, based on the Weighted F1 Score, the Random Forest model was found to handle the class imbalance more effectively than other algorithms. Accordingly, Random Forest was chosen as the final classification model, with the following optimal hyperparameters: n_estimators=100, max_depth=20, min_samples_split=5, min_sample_leaf = 1.

**Table 3:** Model performance evaluation.

Model	Accuracy	Precision	Recall	Macro	Weighted
				F1-Score	F1-Score
RF	0.698	0.622	0.698	0.262	0.624
LightGBM	0.695	0.620	0.696	0.267	0.623
XGBoost	0.694	0.610	0.694	0.248	0.618
SVM	0.696	0.611	0.696	0.245	0.615

Meanwhile, although all F1-related performance metrics are below 0.7, this is expected given the highly imbalanced nature of the dataset. The Macro-F1 Score, which calculates the unweighted average of F1 Scores across all classes, is relatively low (0.262 for the Random Forest model). This occurs because minority classes contribute equally to the Macro-F1 Score despite their small sample size, causing the metric to be dominated by poor performance on rare classes. On the other hand, the Weighted-F1 Score considers the number of samples in each class and gives more weight to the majority classes. Although the Weighted-F1 Score for the Random Forest is below 0.7 (0.624), it still demonstrates that the model effectively balances precision and recall across all classes, handling class imbalance robustly. Therefore, in this study, the Weighted F1-Score is regarded as the primary metric for evaluating the final model performance, while the Macro-F1 Score provides insight into model behavior on minority classes [29,30].

To assess variable importance, SHAP values were computed for the Random Forest model using the Python TreeExplainer function, which implements the TreeSHAP algorithm optimized for tree-based models. TreeSHAP provides an accurate and efficient approach to calculating SHAP values in ensemble decision tree models such as Random Forest, with the advantage of capturing nonlinearity and interactions between variables.

**Fig 4:**
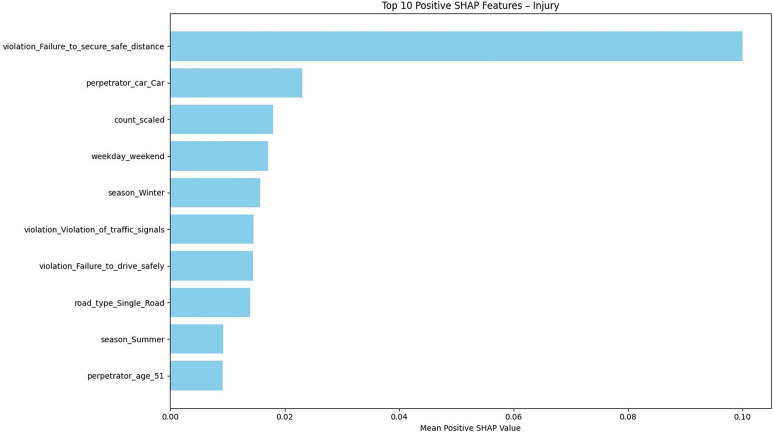
Top 10 Most Influential Features Positively Associated with the Injury Severity Level Based on SHAP Values.

**Fig 5:**
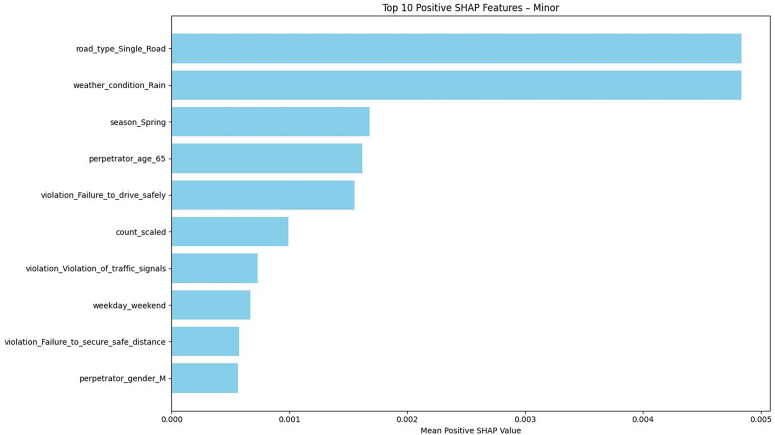
Top 10 Most Influential Features Positively Associated with the Minor Severity Level Based on SHAP Values.

**Fig 6:**
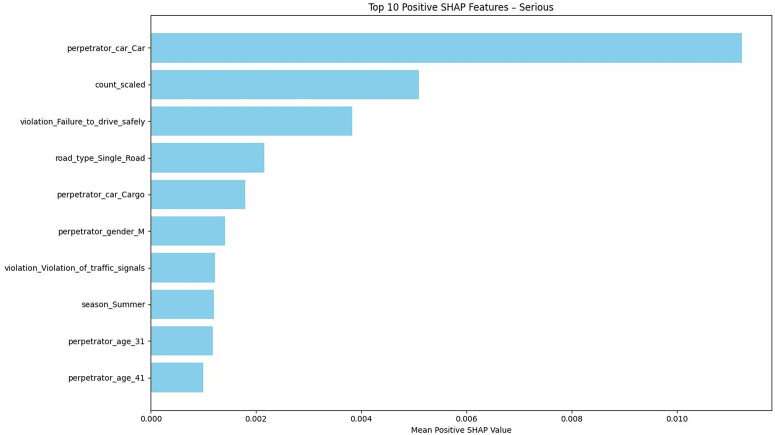
Top 10 Most Influential Features Positively Associated with the Serious Severity Level Based on SHAP Values.

**Fig 7:**
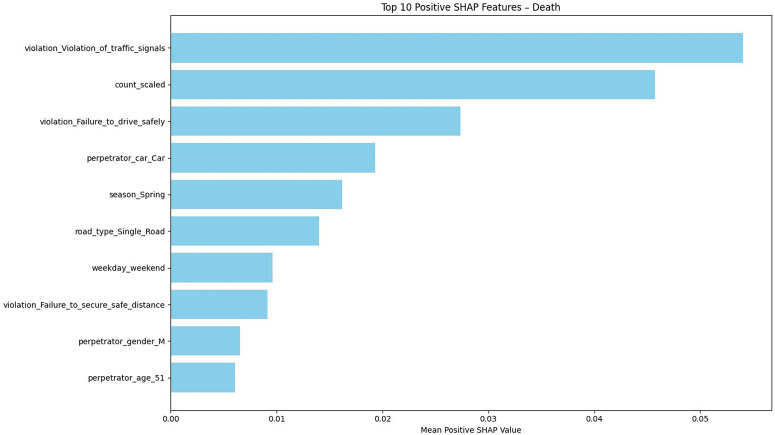
Top 10 Most Influential Features Positively Associated with the Death Severity Level Based on SHAP Values.

In this study, SHAP values were analyzed separately for each level of traffic accident severity (Injury, Minor, Serious, and Death). We focused on positive SHAP values to identify key variables that contribute to increasing the severity of crash. The top 10 variables for each severity level are visualized in Figs 4–7, and the corresponding SHAP values are summarized in S8–S11 Tables, which served as the basis for interpreting the impact of each variable on traffic accident severity.

Fig 4 and S8 Table present the SHAP values corresponding to the ‘Injury’ level of traffic accident severity. According to S8 Table, the most influential variable for the Injury level is ‘violation_Failure_to_secure_safe_distance’, showing the largest positive SHAP contribution to the model prediction of injury-level crash.

Fig 5 and S9 Table show the SHAP values for cases where traffic accident severity is classified as ‘Minor’. Among the top contributing factors, ‘road_type_Single_Road’ and ‘weather_condition_Rain’ exhibit relatively large positive SHAP values, indicating a strong contribution to the model prediction of minor-severity crash.

Single roads, which often lack median barriers or have less clearly separated lanes, may be associated with increased vehicle interactions within the modeled context. Similarly, rainy weather conditions contribute positively to the predicted Minor severity level, reflecting their role in the model’s assessment of traffic accident severity.

In addition, ‘season_Spring’, ‘perpetrator_age_65’, and ‘violation_Failure_to_drive_safely’ also show positive SHAP contributions, suggesting that these factors are associated with the model’s prediction of Minor-level outcomes.

Fig 6 and S10 Table present the SHAP values for crash classified as ‘Serious’. The key contributors at this severity level include ‘perpetrator_car_Car’, ‘count’, ‘violation_Failure_to_drive_safely’, and ‘road_type_Single_Road’.

Among these, ‘perpetrator_car_Car’ exhibits positive SHAP values, indicating that a crash involving passenger cars contribute positively to the model’s prediction of serious crash severity. Similarly, a higher crash ‘count’, unsafe driving behavior (‘violation_Failure_to_drive_safely’), and single-road environments (‘road_type_Single_Road’) are associated with increased predicted severity within the model.

Note that the SHAP values of ‘perpetrator_car_Car’ should be interpreted as reflecting the model-implied relative contribution of passenger-car-related crashes, conditional on other covariates, rather than as an exposure-based or circular effect.

Additionally, higher traffic accident counts and unsafe driving behaviors, represented by violations such as ‘Failure_to_drive_safely’, exhibit consistently positive SHAP contributions, indicating their importance in the model’s assessment of serious traffic accident severity. These patterns suggest that traffic volume and compliance-related factors are salient predictors of severity within the modeling framework. However, interpretation of the ‘count’ variable should be cautious, as it may partially reflect the ordinal severity outcome itself, introducing potential overlap and endogeneity.

Fig 7 and S11 Table present the SHAP values for the ‘Death’ severity level. At this level, ‘violation_Violation_of_traffic_signals’, ‘count’, and ‘violation_Failure_to_drive_safely’ exhibit the largest positive SHAP values, indicating strong contributions to the model’s prediction of death-level traffic accident severity.

In particular, ‘violation_Violation_of_traffic_signals’ shows a substantial positive SHAP contribution, suggesting that traffic signal violations are strongly associated with higher predicted severity within the model. Similarly, a higher crash count and unsafe driving behavior (‘violation_Failure_to_drive_safely’) also contribute positively to the prediction of fatal outcomes.

In addition, ‘perpetrator_car_Car’ exhibits positive SHAP values, indicating that crash involving passenger cars contribute to higher predicted death severity conditional on other covariates included in the model.

The road type (e.g., ‘Single_Road’) and environmental factors, including season (e.g., ‘Spring’) also demonstrated positive SHAP contributions to the model’s prediction of fatal traffic accident severity. This suggests that seasonal conditions and road characteristics play a role in the model’s representation of severity-related patterns.

Overall, the SHAP analysis shows that several violation-related variables (‘Violation_of_traffic_signals’, ‘Failure_to_drive_safely’) and road environment indicators (‘Single_Road’) appear among the top contributing features across multiple severity levels in the model. Note that SHAP values represent model-based contributions to the predicted outcome and do not imply causal relationships or marginal probability effects.

### Integrated findings

To identify robust and consistent risk factors, we compare the findings from the ordinal logistic regression and the Random Forest SHAP analysis across severity levels.

Among the variables examined, ‘Failure_to_drive_safely’ emerges as the most consistently identified risk factor across both frameworks. In the marginal effects analysis, it shows positive effects on Injury and Minor severity levels. In the SHAP analysis, it appears among the top contributing features across Minor, Serious, and Death levels. This cross-framework consistency lends strong empirical support to its role as a key determinant of crash severity and underscores the need for stricter enforcement of safe driving obligations.

Both frameworks also reveal a common pattern of heterogeneity across severity levels. In the marginal effects analysis, Injury, Minor, and Death levels are predominantly influenced by traffic law violations, whereas the Serious level exhibits a distinctly different pattern, being more strongly associated with vehicle type and fog conditions. This heterogeneous pattern is similarly reflected in the SHAP analysis, where vehicle type (‘perpetrator_car_Car’) consistently appears as a key contributor at the Serious severity level. This severity-level heterogeneity, consistently captured across both frameworks, highlights the importance of differentiated policy interventions tailored to each crash severity outcome.

In addition, ‘Single_Road’ emerges as a notable risk factor across multiple severity levels in the SHAP analysis, appearing among the top contributors for Minor, Serious, and Death levels. Although this variable was not explicitly highlighted in the marginal effects analysis, its consistent appearance across multiple severity levels in the SHAP results underscores the policy relevance of road infrastructure characteristics, particularly the absence of median barriers on undivided roads.

## Discussion

Although traffic violations and road types have been widely recognized as key risk factors in traffic safety research, the contribution of this study does not lie in identifying previously unknown variables. Rather, it lies in providing an integrated and interpretable assessment of the relative importance and effect directions of well-established factors through the complementary use of statistical modeling and machine learning approaches. By applying ordinal logistic regression alongside multiple machine learning models, we identify risk factors that are consistently important across different modeling frameworks. This joint interpretation goes beyond performance-oriented analyses and offers a more robust and nuanced understanding of traffic accident severity, particularly in a mid-sized urban–rural context, thereby reinforcing and contextualizing existing knowledge in a policy-relevant manner. By leveraging empirical data specific to Cheongju, our research provides regionally tailored insights—a contribution that distinguishes our work from prior studies with broader or less localized focus.

Our findings highlight the critical influence of frequent violations—such as unsafe driving behaviors and disobedience of traffic signals—on traffic accident severity. This empirical evidence underscores the necessity for targeted behavioral interventions, including tailored education programs for elderly drivers and habitual violators, experiential traffic safety training, and enhanced public awareness campaigns.

From a regulatory standpoint, our results underscore the importance of strengthening and prioritizing existing enforcement efforts, particularly for high-risk violations such as signal violations and failures to drive safely. Rather than advocating entirely new regulatory instruments, the findings support more targeted use of current mechanisms, including automated enforcement systems (e.g., red-light cameras), refined penalty structures, and systematic monitoring of repeat offenders. In terms of infrastructure, the analysis indicates that traffic accident severity is consistently higher on undivided single roads, suggesting that such road types should be considered priority locations for context-specific safety management. Overall, these policy-relevant implications do not introduce novel interventions, but provide empirical evidence to refine and contextualize existing traffic safety strategies.

This study has several limitations. First, the traffic accident severity variable consists of imbalanced data with a very low proportion of fatal crash, which may limit the predictive performance of machine learning models in minority classes. This study prioritizes interpretability and preservation of the true occurrence distribution, focusing on raw data-based analyses. Additional analyses using sampling techniques are presented as supplementary data.

Second, because this study utilized administrative data based on police reports, potential reporting biases may exist, such as underreporting of minor crash, subjective categorization of crash causes, and incomplete variable records. In addition, the granularity of several categorical variables was constrained by data availability and sample size considerations. Age categories were defined to ensure sufficient observations within each group, as finer stratification—particularly at older ages—would have led to sparse data and unstable estimates. Similarly, heterogeneity within road types and high-resolution meteorological conditions (e.g., lane configuration, rainfall intensity, visibility) could not be examined due to limitations of the administrative records. Moreover, several well-established determinants of crash severity—such as time of day, lighting conditions, traffic volume, speed limits, intersection control, and detailed infrastructure characteristics—were unavailable in the data, which may result in residual confounding and omitted-variable bias.

Third, some explanatory variables were categorical due to the nature of administrative data, requiring recoding for analysis. This limits variables such as age to reflect continuous information and can only be interpreted at the categorical level. However, these variables were not assumed to be ordinal or continuous, but rather treated as dummy variables (one-hot encoding) to prevent artificial numerical ordering or distance assumptions from influencing the analysis results. Fourth, this study relies on correlation analysis based on observational data, limiting its ability to directly establish a causal relationship between identified factors and traffic accident severity. Future research needs to verify the causal mechanism through more sophisticated data and research designs.

Looking ahead, several methodological and data-related limitations of the present study suggest directions for future research rather than definitive solutions. First, although ordinal logistic regression provided interpretable estimates of effect directions, its capacity to capture complex nonlinear relationships remains limited. The adoption of spline-based ordinal logistic regression may therefore offer a more flexible framework, particularly for modeling threshold effects and nonlinear risk patterns that could not be fully explored in the current analysis.

Second, the analysis relies on correlational associations derived from observational data, which restricts causal interpretation of the estimated effects. Future studies incorporating causal inference frameworks—such as quasi-experimental designs or causal machine learning approaches—would be necessary to disentangle causal mechanisms and to support stronger policy evaluations.

Third, future studies could extend the analysis to examine protective or mitigating factors by exploring negative SHAP contributions and interactions among explanatory variables.

Finally, the scope of available variables constrained the granularity of the analysis. Important dimensions, including detailed crash types, violation–collision pairings, and high-resolution environmental and spatial information, were not available in the current dataset. The inclusion of such information in future data collection efforts would enable more precise modeling of crash mechanisms and support more context-specific and evidence-based policy assessments.

## Conclusion

This study combines statistical modeling and machine learning techniques using Cheongju regional data to explore the primary determinants of traffic accident severity. Our findings suggest that a multi-dimensional intervention strategy—incorporating behavioral education, enforcement measures, and technological/infrastructural enhancements—may help reduce both the occurrence and severity of traffic crash.

Moreover, our proposed methodological and data enhancements, including spline-based regression models, the use of causal inference techniques, and the integration of more granular environmental and behavioral variables, point to promising directions for future research. These advancements could improve analytic precision and enhance the relevance of findings for evidence-based traffic safety policy development.

## Supporting information

S1 Table Description of variables. (DOCX)

S2 Table Generalized Variance Inflation Factor (GVIF). (DOCX)

S3 Table The result of the association analysis. (DOCX)

S4 Table Average marginal effects from ordinal logistic regression. (DOCX)

S5 Table Ordinal Logistic Regression Result. (DOCX)

S6 Table Tuning parameter performance optimization (with sampling). The bold values mean optimized parameters.
(DOCX)

S7 Table Model performance evaluation (with sampling). (DOCX)

S8 Table Top 10 levels of explanatory variables with positive SHAP values for ‘Injury’ severity level. (DOCX)

S9 Table Top 10 levels of explanatory variables with positive average SHAP values for ‘Minor’ severity level. (DOCX)

S10 Table Top 10 levels of explanatory variables with positive average SHAP values for ‘Serious’ severity level. (DOCX)

S11 Table Top 10 levels of explanatory variables with positive average SHAP values for ‘Death’ severity level. (DOCX)

S1 Data Traffic crash dataset from Cheongju (2019–2023). Police-reported traffic crash records sourced from the Traffic Accident Analysis System (TAAS) operated by the Korea Road Traffic Authority.
(CSV)
