# Supplementary material for: Identifying policy-relevant traffic crash risk factors in Cheongju, South Korea using logistic regression and explainable machine learning
Source: PLoS One. 2026 Jun 22;21(6):e0350616. doi: 10.1371/journal.pone.0350616 (PMC13286193; doi:10.1371/journal.pone.0350616)
Supplement: S1 Table — (DOCX) [file pone.0350616.s001.docx]

**Supplementary Table S1.** Description of variables

| **Description of variables** | | | | |
| --- | --- | --- | --- | --- |
| **Variable** | | **Type** | **Description** | **Descriptive statistic** |
| **Response Variable** | | | | |
| *severity* | | Categorical | 1: Injury | 503 (2.64%) |
|  |  |  | 2: Minor | 13,438 (70.46%) |
|  |  |  | 3: Serious | 4,924 (25.82%) |
|  |  |  | 4: Death | 206 (1.08%) |
| **Explanatory Variables** | | | | |
| Accident  factors | *count* | Continuous | The number of casualties in the event of an accident | Min/Q1/Median/Mean(sd)/Q3/Max |
|  |  |  |  | 1 / 1 / 1 / 1.545(1.104) / 2 / 26 |
|  | *violation* | Categorical | 1: Crossing_center_line | 734 (3.85%) |
|  |  |  | 2: Failure_to_drive_safely | 10,466 (54.88%) |
|  |  |  | 3: Failure_to_secure_safe_distance | 1,737 (9.11%) |
|  |  |  | 4: Illegal_U-turn | 121 (0.63%) |
|  |  |  | 5: Lane_violation | 385 (2.02%) |
|  |  |  | 6: Obstruction_of_straight_right_turn | 507 (2.66%) |
|  |  |  | 7: Violation_of_traffic_signals | 2,613 (13.70%) |
|  |  |  | 8: Violation_of_pedestrian_protection_duty | 558 (2.93%) |
|  |  |  | 9: Violation_of_intersection_driving_method | 1,950 (10.22%) |
| Environmental factors | *season* | Categorical | 1: Autumn | 5,117 (26.83%) |
|  |  |  | 2: Spring | 4,761 (24.96%) |
|  |  |  | 3: Summer | 4,943 (25.92%) |
|  |  |  | 4: Winter | 4,250 (22.29%) |
|  | *weekday* | Categorical | 1: Weekday | 14,278 (74.87%) |
|  |  |  | 2: Weekend | 4,793 (25.13%) |
|  | *weather_condition* | Categorical | 1: Clear | 17,077 (89.54%) |
|  |  |  | 2: Cloudy | 617 (3.24%) |
|  |  |  | 3: Fog | 20 (0.11%) |
|  |  |  | 4: Rain | 1,250 (6.55%) |
|  |  |  | 5: Snow | 107 (0.56%) |
| Road  factor | *road_type* | Categorical | 1: Crossroads | 11,922 (62.51%) |
|  |  |  | 2: Single_Road | 7,149 (37.49%) |
| Vehicle  factor | *perpetrator_car* | Categorical | 1: Agricultural Machinery | 23 (0.12%) |
|  |  |  | 2: All-Terrain Vehicle (ATV) | 15 (0.08%) |
|  |  |  | 3: Bicycle | 385 (2.02%) |
|  |  |  | 4: Car | 13,390 (70.21%) |
|  |  |  | 5: Cargo | 2,156 (11.31%) |
|  |  |  | 6: Construction_Machinery | 184 (0.96%) |
|  |  |  | 7: Motorcycle | 227 (1.19%) |
|  |  |  | 8: Personal Mobility (PM) | 167 (0.88%) |
|  |  |  | 9: Special | 115 (0.60%) |
|  |  |  | 10: Two-wheeled | 1,527 (8.01%) |
|  |  |  | 11: Van | 882 (4.62%) |
| Human  factors | *perpetrator_gender* | Categorical | 1: Female (F) | 4,759 (24.95%) |
|  |  |  | 2: Male (M) | 14,312 (75.05%) |
|  | *perpetrator_age* | Categorical | 1: 20 (Less than 20) | 678 (3.56%) |
|  |  |  | 2: 21 (21--30) | 3,192 (16.74%) |
|  |  |  | 3: 31 (31--40) | 3,002 (15.74%) |
|  |  |  | 4: 41 (41--50) | 3,506 (18.38%) |
|  |  |  | 5: 51 (51--60) | 4,273 (22.41%) |
|  |  |  | 6: 61 (61--65) | 1,635 (8.57%) |
|  |  |  | 7: 65 (More than 65) | 2,785 (14.60%) |
