# Supplementary material for: Identifying policy-relevant traffic crash risk factors in Cheongju, South Korea using logistic regression and explainable machine learning
Source: PLoS One. 2026 Jun 22;21(6):e0350616. doi: 10.1371/journal.pone.0350616 (PMC13286193; doi:10.1371/journal.pone.0350616)
Supplement: S2 Table — (DOCX) [file pone.0350616.s002.docx]

**Supplementary Table S2.** Generalized Variance Inflation Factor (GVIF)

| **Variable** | | **GVIF** | **DF** |
| --- | --- | --- | --- |
| Accident factors | *count* | 1.028 | 1 |
|  | *violation* | 1.300 | 8 |
| Environmental factors | *season* | 1.040 | 3 |
|  | *weekday* | 1.016 | 1 |
|  | *weather_condition* | 1.042 | 4 |
| Road factor | *road_type* | 1.253 | 1 |
| Vehicle factor | *perpetrator_car* | 1.421 | 10 |
| Human factors | *perpetrator_gender* | 1.139 | 1 |
|  | *perpetrator_age* | 1.280 | 6 |
