# Supplementary material for: Identifying policy-relevant traffic crash risk factors in Cheongju, South Korea using logistic regression and explainable machine learning
Source: PLoS One. 2026 Jun 22;21(6):e0350616. doi: 10.1371/journal.pone.0350616 (PMC13286193; doi:10.1371/journal.pone.0350616)
Supplement: S3 Table — (DOCX) [file pone.0350616.s003.docx]

**Supplementary Table S3.** The result of the association analysis

| **Variable** | | **Statistic** | **p-value** |
| --- | --- | --- | --- |
| Accident factors | *count* | 54.46 | <0.001 |
|  | *violation* | 657.35 | 0.0005 |
| Environmental factors | *season* | 27.00 | 0.0014 |
|  | *weekday* | 4.00 | 0.2617 |
|  | *weather_condition* | 22.23 | 0.0490 |
| Road factor | *road_type* | 65.54 | <0.001 |
| Vehicle factor | *perpetrator_car* | 1176.90 | 0.0005 |
| Human factors | *perpetrator_gender* | 32.10 | <0.001 |
|  | *perpetrator_age* | 108.40 | 0.0005 |
