# Supplementary material for: Identifying policy-relevant traffic crash risk factors in Cheongju, South Korea using logistic regression and explainable machine learning
Source: PLoS One. 2026 Jun 22;21(6):e0350616. doi: 10.1371/journal.pone.0350616 (PMC13286193; doi:10.1371/journal.pone.0350616)
Supplement: S4 Table — (DOCX) [file pone.0350616.s004.docx]

**Supplementary Table S4.** Average marginal effects from ordinal logistic regression

| **Variable** | **Reference** | **Injury** | **Minor** | **Serious** | **Death** |
| --- | --- | --- | --- | --- | --- |
| *count* | - | -0.004 | -0.026 | 0.028 | 0.002 |
| *perpetrator_age* | |  | | | |
| 21 | 20 | -0.003 | -0.015 | 0.017 | 0.001 |
| 31 |  | -0.006 | -0.031 | 0.035 | 0.002 |
| 41 |  | -0.005 | -0.030 | 0.033 | 0.002 |
| 51 |  | -0.006 | -0.034 | 0.037 | 0.002 |
| 61 |  | -0.005 | -0.025 | 0.028 | 0.002 |
| 65 |  | -0.007 | -0.042 | 0.047 | 0.003 |
| *Perpetrator_car* | |  | | | |
| ATV | Agricultural  Machinery | -0.004 | -0.207 | 0.150 | 0.062 |
| Bicycle |  | 0.012 | 0.224 | -0.211 | -0.024 |
| Car |  | 0.021 | 0.296 | -0.288 | -0.029 |
| Cargo |  | 0.014 | 0.245 | -0.233 | -0.026 |
| Construction Machinery |  | 0.010 | 0.200 | -0.187 | -0.023 |
| Motocycle |  | 0.012 | 0.227 | -0.214 | -0.025 |
| PM |  | 0.016 | 0.263 | -0.251 | -0.027 |
| Special |  | 0.014 | 0.247 | -0.235 | -0.026 |
| Two-wheeled |  | 0.020 | 0.288 | -0.279 | -0.029 |
| Van |  | 0.016 | 0.260 | -0.249 | -0.027 |
| *Perpetrator_*gender | Female | -0.001 | -0.007 | 0.007 | 0.001 |
| *road_type* | Crossroads | -0.003 | -0.017 | 0.018 | 0.001 |
| *season* | |  | | | |
| Spring | Autumn | -0.002 | -0.017 | 0.018 | 0.002 |
| Summer |  | 0.001 | 0.007 | -0.008 | -0.001 |
| Winter |  | 0.002 | 0.011 | -0.012 | -0.001 |
| *violation* | |  | | | |
| Failure to drive safely | Crossing  center line | 0.014 | 0.127 | -0.132 | -0.008 |
| Failure to secure safe distance |  | 0.026 | 0.177 | -0.192 | -0.011 |
| Illegal U turn |  | 0.014 | 0.126 | -0.131 | -0.008 |
| Lane violation |  | 0.010 | 0.101 | -0.212 | -0.012 |
| Obstruction of straight right turn |  | 0.010 | 0.101 | -0.104 | -0.007 |
| Violation of traffic signals |  | -0.001 | -0.012 | 0.012 | 0.001 |
| Violation of intersection driving method |  | 0.012 | 0.119 | -0.123 | -0.008 |
| Violation of pedestrian protection duty |  | -0.005 | -0.105 | 0.101 | 0.010 |
| *weather_condition* | |  | | | |
| Cloudy | Clear | -0.003 | -0.017 | 0.019 | 0.001 |
| Fog |  | -0.017 | -0.203 | 0.202 | 0.018 |
| Rain |  | -0.002 | -0.012 | 0.013 | 0.001 |
| Snow |  | 0.017 | 0.068 | -0.080 | -0.004 |
| *weekday* | weekday | 0.002 | 0.002 | -0.012 | -0.001 |
