# Supplementary material for: Identifying policy-relevant traffic crash risk factors in Cheongju, South Korea using logistic regression and explainable machine learning
Source: PLoS One. 2026 Jun 22;21(6):e0350616. doi: 10.1371/journal.pone.0350616 (PMC13286193; doi:10.1371/journal.pone.0350616)
Supplement: S5 Table — (DOCX) [file pone.0350616.s005.docx]

**Supplementary Table S5.** Ordinal Logistic Regression Result

| **Variable** | | **Coefficient** | **Odds Ratio** | **Std. Error** | **z-value** | **p-value** |
| --- | --- | --- | --- | --- | --- | --- |
| *Weekday* | weekend | -0.0658 | 0.9363 | 0.0374 | -1.7592 | 0.7857 |
| *violation* | Failure to drive safely | -0.6752 | 0.5091 | 0.0783 | -8.6266 | <2e-16 |
|  | Failure to secure safe distance | -1.0578 | 0.3472 | 0.0942 | -11.2325 | <2e-16 |
|  | Illegal U turn | -0.6700 | 0.5118 | 0.2168 | -3.0893 | 0.00201 |
|  | Lane violation | -1.2138 | 0.2971 | 0.1442 | -8.4184 | <2e-16 |
|  | Obstruction of straight right turn | -0.5143 | 0.5979 | 0.1248 | -4.1215 | 3.76e-05 |
|  | Violation of traffic signals | 0.0568 | 1.0585 | 0.0875 | 0.6499 | 0.51582 |
|  | Violation of intersection driving method | 0.4580 | 1.5809 | 0.1137 | 4.0281 | 5.63e-05 |
|  | Violation of pedestrian protection duty | -0.6235 | 0.5361 | 0.0932 | -6.6890 | 2.25e-11 |
| *weather* | Cloudy | 0.1037 | 1.1092 | 0.0898 | 1.1544 | 0.24831 |
|  | Fog | 0.9950 | 2.7047 | 0.4520 | 2.2013 | 0.02772 |
|  | Rain | 0.0747 | 1.0776 | 0.0646 | 1.1571 | 0.24720 |
|  | Snow | -0.5066 | 0.6025 | 0.2373 | -2.1351 | 0.03275 |
| *Road type* | Single Road | 0.1023 | 1.1077 | 0.0376 | 2.7203 | 0.00652 |
| *Perpetrator_car* | ATV | 1.0278 | 2.7948 | 0.7536 | 1.3639 | 0.17084 |
|  | Bicycle | -1.0070 | 0.3653 | 0.4734 | -2.1271 | 0.03405 |
|  | Car | -1.4149 | 0.2430 | 0.4592 | -3.0815 | 0.00211 |
|  | Cargo | -1.1179 | 0.3270 | 0.4607 | -2.4266 | 0.01557 |
|  | Construction Machinery | -0.8898 | 0.4107 | 0.4836 | -1.8399 | 0.06690 |
|  | Motocycle | -1.0225 | 0.6897 | 0.4812 | -2.1250 | 0.03422 |
|  | PM | -1.2147 | 0.2968 | 0.4953 | -2.4494 | 0.01460 |
|  | Special | -1.1298 | 0.3231 | 0.5010 | -2.2550 | 0.02459 |
|  | Two-wheeled | -1.3660 | 0.2551 | 0.4629 | -2.9507 | 0.00325 |
|  | Van | -1.2017 | 0.3007 | 0.4642 | -2.5886 | 0.00985 |
| Perpetrator_gender | Male | 0.0405 | 1.0413 | 0.0397 | 1.0201 | 0.30760 |
| Perpetrator_age | 21 | 0.1017 | 1.1070 | 0.1008 | 1.0087 | 0.31294 |
|  | 31 | 0.2032 | 1.2253 | 0.1019 | 1.9949 | 0.04601 |
|  | 41 | 0.1946 | 1.2149 | 0.1016 | 1.9158 | 0.05534 |
|  | 51 | .02171 | 1.2425 | 0.1008 | 2.1536 | 0.03124 |
|  | 61 | 0.1628 | 1.1768 | 0.1097 | 1.4833 | 0.13788 |
|  | 65 | 0.2682 | 1.3077 | 0.1027 | 2.6113 | 0.00901 |
| Season | Spring | 0.1018 | 1.1071 | 0.0441 | 2.3057 | 0.02113 |
|  | Summer | -0.0465 | 0.9546 | 0.0444 | -1.0467 | 0.29521 |
|  | Winter | -0.0659 | 1.1071 | 0.0464 | -1.4193 | 0.15580 |
| count | - | 0.1575 | 1.1706 | 0.0140 | 11.2450 | <2e-16 |
| intercept | Injury\|Minor | -5.0943 | 0.0061 | 0.4799 | -10.6149 | 3.57e-07 |
|  | Minor\|Serious | -0.3697 | 0.6909 | 0.4777 | -0.7740 | 0.714365 |
|  | Serious\|Death | 3.2169 | 24.9507 | 0.4818 | 6.6769 | 0.001279 |
