# Supplementary material for: Identifying policy-relevant traffic crash risk factors in Cheongju, South Korea using logistic regression and explainable machine learning
Source: PLoS One. 2026 Jun 22;21(6):e0350616. doi: 10.1371/journal.pone.0350616 (PMC13286193; doi:10.1371/journal.pone.0350616)
Supplement: S6 Table — The bold values mean optimized parameters. (DOCX) [file pone.0350616.s006.docx]

**Supplementary Table S6.** Tuning parameter performance optimization (with sampling). The bold values mean optimized parameters.

| **Algorithm** | **Parameter** | **Value** |
| --- | --- | --- |
| SVM | C | 0.1, 1, **10** |
|  | kernel | linear, **rbf** |
|  | gamma | **scale**, auto |
| *RF* | n_estimators | 50, 100, 200, **500** |
|  | max_depth | 3, 5, 10, **20** |
|  | min_samples_split | 2, **5**, 10 |
|  | min_sample_leaf | **1**, 2, 4 |
| *XGBoost* | n_estimators | 50, 100, 200, **500** |
|  | max_depth | 3, **5**, 10, 20 |
|  | learning_rate | 0.01, 0.05, 0.1, **0.2** |
|  | subsample | 0.4, 0.6, 0.8, **1.0** |
| *LightGBM* | n_estimators | 50, 100, 200, **500** |
|  | max_depth | 3, 5, **10**, 20 |
|  | learning_rate | 0.01, 0.05, **0.1** |
|  | num_leaves | 20, **30**, 40 |
|  | subsample | **0.6**, 0.8, 1.0 |
|  | colsample_bytree | **0.6**, 0.8, 1.0 |
