# Supplementary material for: Identifying policy-relevant traffic crash risk factors in Cheongju, South Korea using logistic regression and explainable machine learning
Source: PLoS One. 2026 Jun 22;21(6):e0350616. doi: 10.1371/journal.pone.0350616 (PMC13286193; doi:10.1371/journal.pone.0350616)
Supplement: S7 Table — (DOCX) [file pone.0350616.s007.docx]

**Supplementary Table S7.** Model performance evaluation (with sampling)

| **Model** | **Accuracy** | **Precision** | **Recall** | **Macro F1-Score** | **Weighted F1-Score** |
| --- | --- | --- | --- | --- | --- |
| *RF* | 0.573 | 0.619 | 0.573 | 0.307 | 0.593 |
| *LightGBM* | 0.552 | 0.612 | 0.552 | 0.293 | 0.578 |
| *XGBoost* | 0.570 | 0.620 | 0.570 | 0.305 | 0.592 |
| *SVM* | 0.557 | 0.628 | 0.557 | 0.298 | 0.588 |
