# Supplementary material for: Identifying policy-relevant traffic crash risk factors in Cheongju, South Korea using logistic regression and explainable machine learning
Source: PLoS One. 2026 Jun 22;21(6):e0350616. doi: 10.1371/journal.pone.0350616 (PMC13286193; doi:10.1371/journal.pone.0350616)
Supplement: S8 Table — (DOCX) [file pone.0350616.s008.docx]

**Supplementary Table S8.** Top 10 levels of explanatory variables with positive SHAP values for ‘Injury’ severity level

| **Explanatory variable** | **Level of explanatory variable** | **SHAP value** |
| --- | --- | --- |
| *violation* | Failure to secure safe distance | 0.100027 |
| *perpetrator_car* | Car | 0.023102 |
| *count* | - | 0.017949 |
| *weekday* | weekend | 0.017132 |
| *season* | Winter | 0.015692 |
| *violation* | Violation of traffic signals | 0.014568 |
| *violation* | Failure to drive safely | 0.014427 |
| *road_type* | Single Road | 0.013995 |
| *season* | Summer | 0.009300 |
| *perpetrator_age* | 51 | 0.009127 |
