# Supplementary material for: Identifying policy-relevant traffic crash risk factors in Cheongju, South Korea using logistic regression and explainable machine learning
Source: PLoS One. 2026 Jun 22;21(6):e0350616. doi: 10.1371/journal.pone.0350616 (PMC13286193; doi:10.1371/journal.pone.0350616)
Supplement: S9 Table — (DOCX) [file pone.0350616.s009.docx]

**Supplementary Table S9.** Top 10 levels of explanatory variables with positive average SHAP values for ‘Minor’ severity level

| **Explanatory variable** | **Level of explanatory variable** | **SHAP value** |
| --- | --- | --- |
| *road_type* | Single Road | 0.004837 |
| *weather_condition* | Rain | 0.004836 |
| *season* | Spring | 0.001679 |
| *perpetrator_age* | 65 | 0.001618 |
| *violation* | Failure to drive safely | 0.001553 |
| *count* | - | 0.000990 |
| *violation* | Violation of traffic signals | 0.000727 |
| *weekday* | weekend | 0.000666 |
| *violation* | Failure to secure safe distance | 0.000575 |
| *perpetrator_gender* | Male | 0.000563 |
