# Supplementary material for: Identifying policy-relevant traffic crash risk factors in Cheongju, South Korea using logistic regression and explainable machine learning
Source: PLoS One. 2026 Jun 22;21(6):e0350616. doi: 10.1371/journal.pone.0350616 (PMC13286193; doi:10.1371/journal.pone.0350616)
Supplement: S10 Table — (DOCX) [file pone.0350616.s010.docx]

**Supplementary Table S10.** Top 10 levels of explanatory variables with positive average SHAP values for ‘Serious’ severity level

| **Explanatory variable** | **Level of explanatory variable** | **SHAP value** |
| --- | --- | --- |
| *perpetrator_car* | Car | 0.011241 |
| *count* | - | 0.005100 |
| *violation* | Failure to drive safely | 0.003830 |
| *road_type* | Single Road | 0.002158 |
| *perpetrator_car* | Cargo | 0.001795 |
| *perpetrator_gender* | Male | 0.001414 |
| *violation* | Violation of traffic signals | 0.001216 |
| *season* | summer | 0.001198 |
| *perpetrator_age* | 31 | 0.001181 |
| *perpetrator_age* | 41 | 0.000999 |
