# Supplementary material for: Identifying policy-relevant traffic crash risk factors in Cheongju, South Korea using logistic regression and explainable machine learning
Source: PLoS One. 2026 Jun 22;21(6):e0350616. doi: 10.1371/journal.pone.0350616 (PMC13286193; doi:10.1371/journal.pone.0350616)
Supplement: S11 Table — (DOCX) [file pone.0350616.s011.docx]

**Supplementary Table S11.** Top 10 levels of explanatory variables with positive average SHAP values for ‘Death’ severity level

| **Explanatory variable** | **Level of explanatory variable** | **SHAP value** |
| --- | --- | --- |
| *violation* | Violation of traffic signals | 0.054054 |
| *count* | - | 0.045759 |
| *violation* | Failure to drive safely | 0.027350 |
| *perpetrator_car* | Car | 0.019306 |
| *season* | Spring | 0.016176 |
| *road_type* | Single Road | 0.014003 |
| *weekday* | weekdend | 0.009602 |
| *violation* | Failure to secure safe distance | 0.009153 |
| *perpetrator_gender* | Male | 0.006558 |
| *perpetrator_age* | 51 | 0.006058 |
